# Detecting Emerging Transmissibility of Avian Influenza Virus in Human Households

**DOI:** 10.1371/journal.pcbi.0030145

**Published:** 2007-07-27

**Authors:** Michiel van Boven, Marion Koopmans, Mirna Du Ry van Beest Holle, Adam Meijer, Don Klinkenberg, Christl A Donnelly, Hans (J. A. P.) Heesterbeek

**Affiliations:** 1 Faculty of Veterinary Medicine, Utrecht University, Utrecht, The Netherlands; 2 Animal Sciences Group, Wageningen University and Research Centre, Lelystad, The Netherlands; 3 Centre for Infectious Disease Control, National Institute for Public Health and the Environment, Bilthoven, The Netherlands; 4 Department of Infectious Disease Epidemiology, Imperial College London, London, United Kingdom; Yale University, United States of America

## Abstract

Accumulating infections of highly pathogenic H5N1 avian influenza in humans underlines the need to track the ability of these viruses to spread among humans. A human-transmissible avian influenza virus is expected to cause clusters of infections in humans living in close contact. Therefore, epidemiological analysis of infection clusters in human households is of key importance. Infection clusters may arise from transmission events from (i) the animal reservoir, (ii) humans who were infected by animals (primary human-to-human transmission), or (iii) humans who were infected by humans (secondary human-to-human transmission). Here we propose a method of analysing household infection data to detect changes in the transmissibility of avian influenza viruses in humans at an early stage. The method is applied to an outbreak of H7N7 avian influenza virus in The Netherlands that was the cause of more than 30 human-to-human transmission events. The analyses indicate that secondary human-to-human transmission is plausible for the Dutch household infection data. Based on the estimates of the within-household transmission parameters, we evaluate the effectiveness of antiviral prophylaxis, and conclude that it is unlikely that all household infections can be prevented with current antiviral drugs. We discuss the applicability of our method for the detection of emerging human-to-human transmission of avian influenza viruses in particular, and for the analysis of within-household infection data in general.

## Author Summary

Recent outbreaks of emerging diseases such as SARS and H5N1 avian influenza have underlined the fact that animal pathogens may acquire the ability to spread efficiently in humans. Monitoring the transmissibility of pathogens from the animal reservoir in humans is key for early detection of epidemic spread, and for effective control. Here we have used data from a small but well-defined study of H7N7 avian influenza virus transmission in human households to estimate the transmissibility of H7N7 avian influenza in humans living in close contact. The analyses make use of household final size data (i.e., the number of individuals in the household who are ultimately infected), which, for many pathogens, are easily collected. For the H7N7 data, the analyses indicate that the transmission chain in humans may well have extended beyond the first generation of infections in humans, and that less than half of the household infections could have been prevented by antiviral prophylaxis. Our method of analysis provides a rapid and generally applicable tool that can be used to monitor emerging human transmissibility of pathogens from the animal reservoir.

## Introduction

Outbreaks of highly pathogenic H5N1 avian influenza in Southeast Asia, Europe, and Africa have devastating consequences for poultry [1,2], and have resulted in numerous infections in humans [3–5]. Although these infections from the animal reservoir continue to accumulate, the virus does not seem to spread extensively among humans. Nevertheless, a fear is that these human infections may ultimately spark an influenza pandemic [6–9]. Indeed, recent clusters of infections in human households hint at the possibility of virus transmission from humans who were infected by poultry to their household contacts [10,11]. These suggestions are strengthened by the observation of mutations in recent H5N1 viruses that seem to predispose the virus for more efficient transmission in mammals, including humans [12–16] (but see [17–18]).

It is likely that a virus with pandemic potential will present itself initially through an increase in the number of infections in humans who have been in close contact with the case infected by animals. Therefore, rapid detection and control of clusters of infections is of key importance [7,9]. Such clusters may result from (i) multiple introductions from the animal reservoir (zoonotic transmission), (ii) multiple transmission events from humans who were infected by animals (primary human-to-human transmission), or (iii) multiple transmission events from humans who were themselves infected by humans (secondary human-to-human transmission). Obviously, evidence for (iii) is the most worrisome as it indicates that the virus has acquired the ability to spread efficiently in humans.

It is often thought that pathogens from the animal reservoir that have made the jump to a new host species are usually not (yet) well-adapted for sustained transmission in the new host, and that transmissibility in a new species will gradually increase over time by the process of adaptation by means of natural selection [19–23]. Interestingly, however, in the case of H5N1 avian influenza in humans, the evidence so far does not seem to fit this prediction [22–24]. Mechanisms that could be responsible for the lack of efficient secondary human-to-human transmission could be due to a dose effect whereby humans infected by animals receive a higher infection dose than humans infected by humans, or to behavioural changes after infection that limit spread of the virus after it has been detected.

In this paper we develop a method to detect and quantify different routes of virus transmission in a household setting. Our main aim is to investigate whether within-household pathogen transmission has been restricted to transmission from the primary infected individual or whether there is evidence that the transmission chain has extended beyond the first generation of human-to-human infections. Our analyses are based on theoretical developments on the distribution of the final size of an epidemic in finite populations, which allow construction of flexible methods to analyse within-household transmission chains.

We apply the method to a recent study of within-household transmission of highly pathogenic avian influenza of the H7N7 subtype that caused a large epidemic in poultry in The Netherlands in 2003. Shortly after the detection of virus circulation, the Dutch authorities undertook an aggressive control strategy that consisted of an animal movement ban in the affected regions, tracing and screening suspected flocks, and culling of infected and contiguous flocks. In all, a total of 255 flocks became infected during a period of nine weeks, and more than 30 million birds were culled [25,26]. Subsequent studies of poultry workers revealed that at least 86 infections from the animal reservoir to humans had taken place [27–29]. In addition, more than 30 household contacts of the infected poultry workers who had not been in direct contact with poultry were reported positive. These reports indicate that human-to-human transmission did occur from individuals infected from the animal reservoir.

Here we analyse data of the transmission chains in 24 households that led to 33 human-to-human transmission events, measuring the extent of onward transmission from humans who were infected by humans (i.e., secondary human-to-human transmission). We complement the statistical analyses by systematic (post-hoc) power analyses to obtain insight into the study size needed to be able to find significant secondary human-to-human transmission, given that it is present.

Although we have applied the method to a specific dataset, we believe that our method is of general interest as it enables rapid estimation of within-household transmission rates based on data that are easily gathered for most infectious diseases. For instance, our method of analysis is not restricted to the analysis of emerging pandemic influenza, but it can just as well be used to estimate different routes of within-household transmission rates of human influenza A viruses [30–32] and, importantly, to assess the potential effectiveness of control measures.

## Materials and Methods

### Data

Based on evidence of human-to-human transmission of H7N7 avian influenza virus that was the cause of the outbreak among poultry in The Netherlands [27], a retrospective cohort study was undertaken to determine the extent of human-to-human transmission in households of infected poultry workers [28–29]. Briefly, the families of 63 of 86 poultry workers who were found positive agreed to participate in the study. Of these, 39 households were excluded because direct contact of the household members with infected birds could not be ruled out. Our dataset thus contained 24 households with a single confirmed H7N7 infected poultry worker. There were no indications of an age bias or sex bias in our study population, and the distribution of household sizes in our study was not untypical for the Dutch population [29]. In total, 33 of 56 household members of individuals who were classified as an index case had antibodies to H7N7 virus (Table 1), in contrast with a group of recently vaccinated age-matched and region-matched controls who were all seronegative [33]. For most of the individuals who were classified as positive, the main symptom of infection was conjunctivitis (an infection of the eye), which may have been the point of entry and site of virus multiplication [27–29].

**Table 1:**
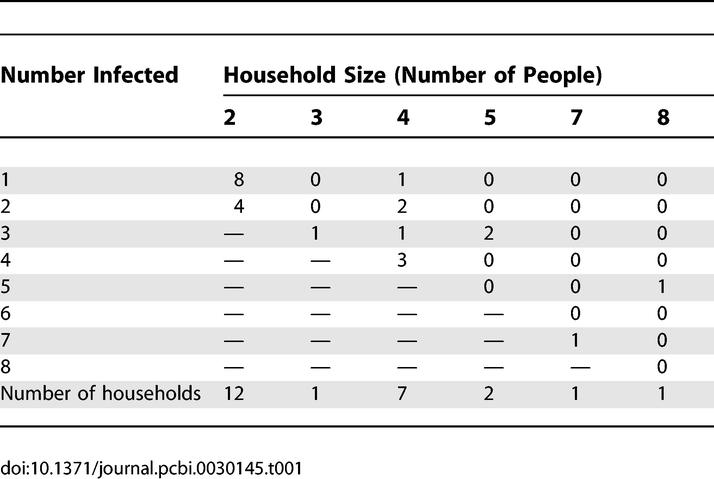
Overview of the Household Final Size Infection Data of Highly Pathogenic H7N7 Avian Influenza Virus That Caused a Major Outbreak among Poultry in The Netherlands in 2003

### Final Size Distribution

The statistical analyses are based on the distributions of the final size (the total number infected in a household) as a function of the household size [34–35], obtained using an SEIR epidemiological model in which individuals are classified as susceptible (S), latently infected (infected but not yet infectious) (E), infectious (I), and recovered and immune (R). No a priori assumptions are made regarding the duration or distribution of the latent and infectious periods. We consider a model with two types of individuals, and assume that there are no individuals who have prior immunity (a plausible assumption for an emerging pathogen). The mathematical equations determining the distribution of the final size of the household outbreaks are given in Text S1.

The final size equation in Text S1 is quite general. The data of the Dutch epidemiological study are more specific and allow a number of simplifications in the final size equation. In particular, the epidemiological study focused on households in which only the initially infected individual had acquired the infection from the animal reservoir, while the other household members had had no contact with infected poultry. We assume that type 1 individuals are infected from the animal reservoir and that type 2 individuals are susceptible to infection by humans. The focus in our analyses is on 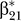 , the transmission rate parameter from the person infected from the animal reservoir to its household members (primary human-to-human transmission), and 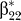 , the transmission rate parameter from humans who are infected by humans (secondary human-to-human transmission).

### Scenarios

In the analyses, we consider four scenarios that are defined by the assumptions regarding the distribution of the infectious period and the mechanism of pathogen transmission. With respect to the infectious period, we focus on two extremes, one in which the infectious period is exponentially distributed (the “general stochastic epidemic”) and one in which the infectious period is of fixed duration (the “Reed-Frost” model) [34–36].

With respect to the mechanism of virus transmission within the household, we assume that transmission is frequency-dependent or density-dependent [37]. In a frequency-dependent model, the number of contacts per unit of time is fixed, and the transmission rate is proportional to the relative frequency (prevalence) of infectious individuals. In a density-dependent model, the number of contacts per unit of time is proportional to the number of individuals. Hence, in a frequency-dependent model, the transmission rate in a household of two individuals of which one is infectious equals the transmission rate in a household of four of which two are infectious. In a density-dependent model, the transmission rate in the latter household would be twice as high as in the former. Notice that the dimension of the transmission parameter 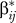 of the density-dependent model is defined per individual per unit time, while the transmission parameter *β_ij_* of the frequency-dependent model is defined per unit time. Notice furthermore that *β_ij_* can be interpreted as the expected number of type *i* infections that would be caused by a type *j* infected individual over the course of its infectious period in a large population of susceptibles if time is measured in units of the infectious period [38]. Further details are given Text S1.

### Statistical Analysis and Model Selection

With the computed final size distributions and household final size data at hand, it is straightforward to estimate the parameters of interest by means of maximum likelihood [30–32], and to calculate the corresponding confidence intervals/areas on the basis of likelihood ratio tests.

To evaluate whether secondary human-to-human transmission has taken place, and to choose between models of different complexity, we make use of the Akaike Information Criterion adjusted for small sample size (AIC_c_) [39]. We focus on a set of one-parameter models with no secondary human-to-human transmission (i.e., 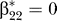 or *β*_22_ = 0), a set of one-parameter models with no difference between primary and secondary human-to-human transmission (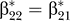 or *β*_22_ = *β*_21_), and a set of two-parameter models in which both transmission parameters are estimated.

The difference Δ*_i_* = *A/C*_i_ − *A/C*_min_ measures the support for model *i*. In general, the larger Δ*_i_*, the less plausible the model is. The model weights or supports 
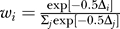
 can be interpreted as the probability that model *i* is the best among the ones considered [39].

### Power Analysis

The size of the Dutch study that inspired our analyses was fairly small (24 households). This may be typical for emerging pathogens from zoonotic origins that have not (yet) acquired the ability to spread efficiently among humans. Therefore, we performed power analyses to evaluate for which effect sizes (i.e., transmission rate parameter values) and study designs (i.e., number of households) secondary human-to-human transmission can be detected by our method. In particular, we carried out post-hoc power analyses of the Dutch epidemiological study taking the estimated parameter values of Table 2, and assuming different sizes of the epidemiological study. In addition, we used simulated datasets in which primary and secondary human-to-human transmission were equally efficient in order to determine the minimal study size that would be necessary for detection of secondary human-to-human transmission.

**Table 2:**
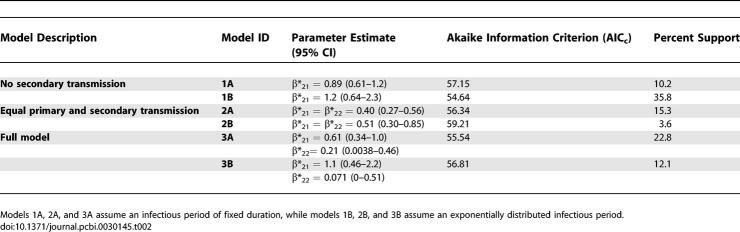
Maximum Likelihood Estimates of the Transmission Rate Parameters of the Models Described in the Text

For each parameter constellation, we carried out 1,000 simulations and re-estimated the parameters of the simulated data as described in the above sections. We focused either on simulated datasets that contained 24 households with the household distribution as in Table 1, or on simulated datasets that contained a multiple of 24 households, while keeping the distribution of household sizes as in Table 1.

### Antiviral Prophylaxis

To evaluate the potential efficacy of prophylactic antiviral drug treatment, we calculate household final size distributions and expected household outbreak sizes if all household members take antiviral drugs. The baseline transmission parameters are as in Table 2. Antiviral drugs reduce the susceptibility of uninfected individuals by a factor 1*-AVE_S_*, where *AVE_S_* is the antiviral efficacy for susceptibility, and the infectiousness of infected individuals by a factor 1*-AVE_I_* where *AVE_I_* is the antiviral efficacy for infectiousness [40–43]. Since all individuals infected from the animal reservoir were already taking antiviral prophylaxis [27–28], the transmission rate parameters 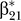 and 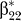 (or 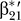 and 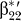 ) in a household that is on antiviral therapy are 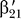 = (1 − *AVE_S_*)*β*_21_ and 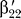 , = (1 – *AVE_I_*)(1 – *AVE_S_*) *β*_22_, respectively. In line with previous studies [9,42–43], we assume in our default scenario that antiviral drug treatment reduces the susceptibility to infection weakly (*AVE_S_* = 0.3), and the infectiousness once infected moderately (*AVE_I_* = 0.6).

A recent study estimated the antiviral efficacies for susceptibility to infection with illness (*AVE_SD_*) and antiviral efficacy for infectiousness at *AVE_SD_* = 0.85 and *AVE_I_* = 0.66 [40–41]. In the Results and Table S4, we therefore also consider scenarios with higher antiviral efficacies for susceptibility and infectiousness. Specifically, we have also considered *AVE_S_* = 0.6 and *AVE_I_* = 0.66, i.e., antiviral drug treatment reduces the overall incidence of infection to a lesser extent than the incidence of symptomatic infection.

## Results

### Estimation of Household Transmission Rates

We consider three scenarios for virus spread within a household after an introduction from the animal reservoir. First, we assume that all household infections are the result of transmission from the person originally infected by the animal reservoir (model 1). Second, we assume that there are no differences in the transmission rates from human cases infected by the animal reservoir and from human cases infected by humans (model 2). Third, we consider a model in which these human-to-human transmission rates are estimated separately (model 3). Within each model we assume that transmission is either frequency-dependent or density-dependent, and that the infectious period is either fixed or highly variable (see Materials and Methods), yielding four scenarios per model (models 1A–1D, models 2A–2D, and models 3A–3D).

**Figure 1:**
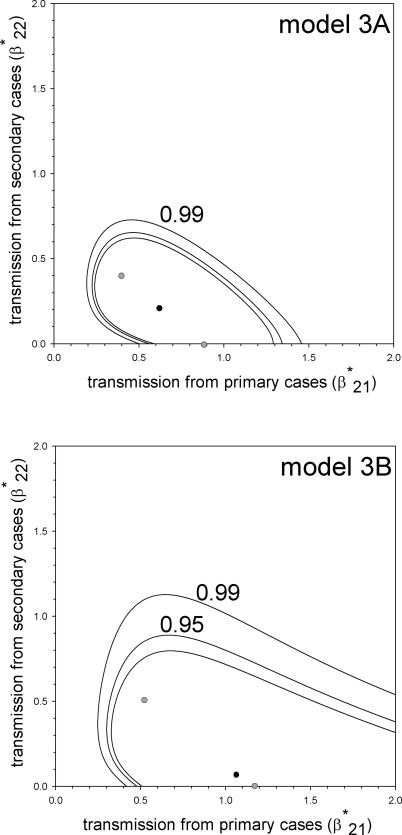
Maximum Likelihood Estimates of the Transmission Rate Parameters for the Model 3A (Top Panel) and Model 3B (Bottom Panel) Described in the Text (Black Dots), with Contours of the 90%, 95%, and 99% Confidence Areas The maximum likelihood parameter estimates of models that exclude secondary human-to-human transmission (β*_22_ = 0, models 1A and 1B), and that assume equal primary and secondary human-to-human transmission (β*_22_ = β*_21_, models 2A and 2B), are also indicated (grey dots).

The results show that there is no single model or scenario that is exclusively favored by the data, although the density-dependent transmission scenarios fit the data considerably better than the frequency-dependent scenarios (Table S1). In fact, the combined support for the density-dependent scenarios is 83.6% versus 16.4% for the frequency-dependent scenarios. Therefore, we will from this point onward focus on the density-dependent scenarios only. The results of the analyses are summarized in Figure 1 and Table 2.

For the model with the highest support (model 1B), the maximum likelihood estimate of the transmission rate parameter for primary human-to-human transmission is 1.2 (95% CI = 0.64–2.3). This implies that the expected numbers of human-to human infections (excluding the primary case) in households of sizes four and eight are 1.6 and 3.8, respectively (Table 3). For the second ranking model (model 3A), the transmission rate parameters for primary and secondary human-to-human transmission are 0.61 (0.34–1.0) and 0.21 (0.0038–0.46), and the expected numbers of human-to-human infections in households of size four and eight are 1.7 and 5.4, respectively.

**Table 3:**
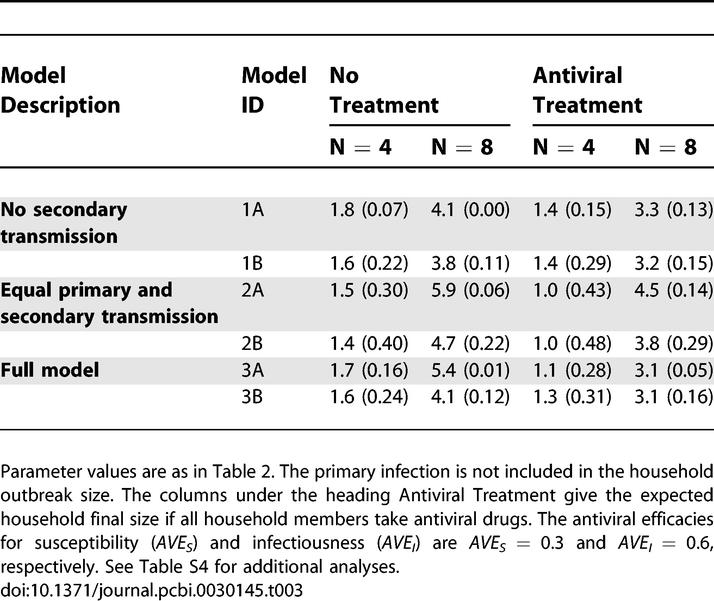
The Expected Outbreak Size and Probability of No Human-to-Human Transmission (Number between Brackets) in Households of Sizes Four and Eight, Respectively

The four models with more than 10% support can be divided in two groups. The first group consists of models 1B and 3B, which have a highly variable infectious period, and no (model 1B) or very little (model 3B) secondary human-to-human transmission. The second group contains models 2A and 3A, which have a fixed infectious period and significant secondary transmission. Apparently, the data are best explained by some differentiation in within-household infectivity, either by probabilistic differences between the primary infecteds (models 1B and 3B) or by differences in infectivity due to the inclusion of secondary human-to-human transmission (models 2A and 3A).

The test that detects H7N7 antibodies in humans has high sensitivity and specificity [30]. Nevertheless, it is possible that the dataset includes a small number of false positives. To investigate this possibility, we reanalysed the data assuming 85% test specificity. Under this assumption, the transmission rate parameters are consistently slightly lower than in Table 2 and Table S1, but the results are otherwise in good agreement with our default scenario (Table S2). In addition, we reanalysed the data when one or both of the large households with a high proportion of test positives are excluded from the analyses (Table 1). The results are similar to those presented in Table 2 and Table S1, the trend being that the frequency-dependent transmission models now have slightly higher support (Table S3).

### Power Analysis

The above results indicate that, on the one hand, secondary human-to-human transmission is plausible if variation in the infectious period is limited, but, on the other hand, may not be necessary if there is substantial variation in the infectious period. Unfortunately, there is to date not enough information to decide which model is more plausible. Therefore, to investigate to what extent our results are a consequence of our small study size, we have carried out a number of power analyses.

**Figure 2:**
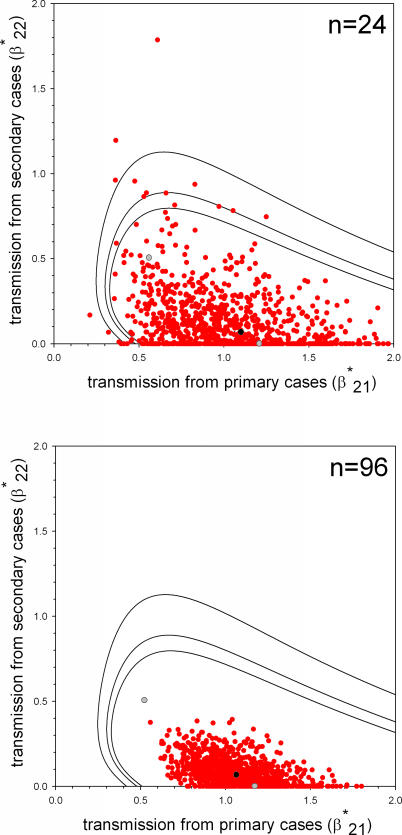
Post-Hoc Power Analysis of Model 3B (i.e., Assuming an Exponentially Distributed Infectious Period) The top panel (*n* = 24) shows the parameter estimates of 1,000 simulated outbreaks in a population of 24 households with size distribution as in Table 1 and with the parameter values as estimated in Table 2. The bottom panel (*n* = 96) shows the results of the analyses in the case that the number of households is increased fourfold.

Figure 2 shows the results of a post-hoc power analysis of model 3B (exponentially distributed infectious period, separate estimation of primary and secondary human-to-human transmission), which yielded a low but positive rate of secondary human-to-human transmission. The top panel shows the point estimates of the transmission rate parameters of 1,000 simulations of a population of 24 households, taking the estimated parameter values of Table 2 (β*_21_ = 1.1 and β*_22_ = 0.071). The analyses show that although 668 of 1,000 simulations yield estimates of the full model with β*_22_ > 0, in the statistical comparison only 263 of the 1,000 simulations favour a model that includes secondary human-to-human transmission. Apparently, the more parsimonious model without secondary human-to-human transmission that contains only one parameter is most of the times favoured over the full model that does include secondary human-to-human transmission but contains two parameters. If the study size is increased from 24 to 96 households, the number of simulations that yield estimates with β*_22_ > 0 increases to 877 of 1,000 (Figure 2, bottom panel). The number of simulations that support models that include secondary human-to-human transmission also increases to 381 of 1,000. If the number of households is increased still further, the support for models that include secondary human-to-human transmission increases still further (unpublished data).

**Figure 3:**
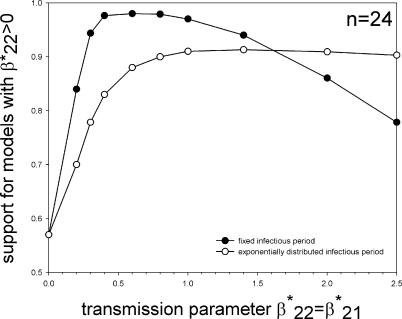
Average Support for Models That Include Secondary Human-to-Human Transmission for 1,000 Simulated Datasets of 24 Households with Equal Primary and Secondary Human-to-Human Transmission (β*_22_ = β*_21_) The ordinate shows the combined support for the one-parameter model with β*_22_ = β*_21_ (model 2) and the two-parameter model in which β*_21_ and β*_22_ are estimated separately (model 3). Black dots refer to simulations with a fixed infectious period, and open dots represents simulations with an exponentially distributed infectious period.

To further investigate the relation between pathogen transmissibility and the ability of our method to distinguish primary from secondary human-to-human transmission in a general setting, we carried out simulations assuming that primary and secondary human-to-human transmission are equally efficient (β*_22_ = β*_21_), and estimated the transmission parameters as described above. The results are summarized in Figure 3. If there is very little human-to-human transmission, there are few infection events in the households, and our method yields low support (57%) for models that include secondary human-to-human transmission (model 1, no secondary transmission: 43%; model 2, equal primary and secondary transmission: 43%; full model: 14%). If the efficiency of human-to-human transmission is increased, the support for models that include secondary human-to-human transmission increases, especially for the model with a fixed infectious period. For the model with a fixed infectious period, the highest support is 98% at β*_22_ = β*_21_ = 0.6 (individual^−1^ * infectious period^−1^), while for the model with an exponentially distributed infectious period it is 91% at β*_22_ = β*_21_ = 1.4 (individual^−1^ * infectious period^−1^). If human-to-human transmission is very efficient, most household members are infected, and the method has difficulties distinguishing between primary and secondary human-to-human transmission. As a consequence, the support for models that include secondary human-to-human transmission decreases with increasing human-to-human transmissibility if human-to-human transmissibility is already efficient.

### Antiviral Prophylaxis

During the Dutch outbreak of H7N7 influenza virus in poultry, prophylactic treatment with the antiviral drug oseltamivir was given to poultry workers but not to their household members. Given the observation of considerable within-household human-to-human transmission, consideration should be given to offering prophylactic treatment with antiviral drugs to all household members of a person at risk of infection. The natural question is to ask how effective such a strategy would have been, given estimates of household transmission parameters (Table 2) and parameters determining the efficacy of antiviral drugs [9,40–43].

For the Dutch study population (Table 1), the expected numbers of household infections for the three most plausible models and parameter estimates of Table 2 are 30.3 (model 1B), 32.7 (model 3A), and 30.1 (model 2A). This corresponds well with the actual number of household infections (33) in the dataset of Table 1. Recalculating the expected number of household infections using conservative estimates of the antiviral efficacies for susceptibility and infectiousness (*AVE_S_ =* 0.3 and *AVE_I_ =* 0.6) [9,42–43] yields expected numbers of household infections of 25.3 (model 1B), 21.5 (model 3A), and 21.8 (model 2A). If antiviral prophylaxis is assumed to be more effective (*AVE_S_ =* 0.6 and *AVE_I_ =* 0.66) [40–41], the expected numbers of household infections are 18.0 (model 1B), 12.9 (model 3A), and 11.7 (model 2A). Hence, for the Dutch study, prophylactic treatment would have been able to prevent 5–11 of the more than 30 household infections if antiviral drugs are moderately efficacious, and 12–20 household infections if the efficacy of antiviral drugs is higher.

Table 3 shows the results of a systematic analysis of the efficacy of prophylactic antiviral treatment for households of four and eight persons. The analyses show that the number of human-to-human infections can be decreased to some extent by targeted antiviral therapy, although it is unlikely that all household infections can be prevented. In Table 3, we have assumed that antiviral drug treatment reduces susceptibility and infectiousness moderately. In Table S4, we investigate the robustness of the results of Table 3 by increasing the antiviral efficacies for susceptibility and infectiousness. The analyses in Table S4 show that the number of household infections decreases with increasing antiviral efficacy for susceptibility or infectiousness. Still, it is unlikely that all household infections can be prevented. We conclude that complete prevention of close-contact infections with current antiviral drugs is probably not within reach, at least not for the H7N7 avian influenza virus that caused the outbreak in poultry in The Netherlands, and which was the cause of dozens of human infections.

## Discussion

We have presented a method to quantify different routes of transmission of avian influenza virus in human households, and applied the method to an epidemiological study that was carried out after a large outbreak of H7N7 avian influenza virus in poultry in The Netherlands. Although the size of the study is relatively small, it is the most detailed investigation of household transmission of avian influenza virus thus far and therefore forms a good starting point to evaluate methods aimed at quantifying human-to-human transmission of avian influenza viruses. Households in which additional bird-to-human transmission could not be ruled out were excluded in order to be able to focus solely on different routes of human-to-human transmission. The methods, however, are also applicable to the situation where the source of the human infections (animal or human) is not known.

The ability of our method to distinguish between primary and secondary human-to-human transmission is determined by the distribution of the final size within households. If secondary human-to-human transmission is efficient (i.e., such that it can lead to a sustained chain of infections in sufficiently large populations of susceptibles), epidemiological theory informs that the final size distribution is expected to be bimodal [38], while if there is no or little secondary human-to-human transmission the final size distribution is expected to remain unimodal (see Text S1 for details). As the power analyses have shown, our method works best for intermediate transmission rates, since then the difference between scenarios with reproduction numbers smaller and higher than 1 is most pronounced. This also explains the difference in Figure 3 between the models, assuming a fixed or exponentially distributed infectious period, as the probability of a minor outbreak is, for a fixed value of the reproduction number, smaller if the infectious period is of fixed duration than if it is exponentially distributed [34]. Given these results, it would be interesting to know the actual distribution of the infectious period of avian influenza viruses in humans. Unfortunately, for avian influenza virus infections in humans, very little is known about the duration and the distribution of the duration of the infectious period. For human influenza viruses, more is known about the duration of the infectious period. For instance, a detailed analysis of H3N2 human influenza virus data indicated that the mean infectious period was about four days, with very little variation around the mean [44]. At present, however, it is unclear if and how these results of human-adapted strains can be translated to strains that are not (yet) adapted for transmission among humans.

Our post-hoc power analyses furthermore indicate that if secondary human-to-human transmission is fairly inefficient (i.e., if β*_22_ is small enough so that no prolonged infection chain is possible in a large population of susceptibles), it can still be detected using fairly small studies that contain a few dozens of households (e.g., *n* = 96 in Figure 2). If, however, secondary human-to-human transmission is very inefficient, larger study sizes are needed, probably containing several hundreds of households, to unequivocally demonstrate the existence of secondary human-to-human transmission. In other words, our method is well-suited to detect animal pathogens that are on the verge of obtaining the ability for continued spread in humans.

Our general power analyses in Figure 3 also have shown that our model is able to detect efficient secondary human-to-human transmission with very small study sizes (e.g., *n* = 24 in Figure 3). Perhaps surprisingly, the method works best when transmission rates are intermediate, because then the difference between models that include or exclude secondary human-to-human transmission is most pronounced. It would be interesting to investigate whether these types of phenomena are also observed in other and more general two-type epidemic models for the spread of pathogens within households [21–23].

For the Dutch outbreak, our results show that there is some but no conclusive evidence of secondary human-to-human virus transmission. In fact, the combined support for the four models that exclude secondary human-to-human transmission (models 1A–1D) is 39.8%, while the combined support for the eight models that do include secondary human-to-human transmission (models 2A–2D and 3A–3D) is only 60.2% (Table S1). In addition, the model with the highest individual support (model 1B) has a support of 29.9% and does not include secondary transmission (Table S1). Moreover, if both transmission parameters are estimated separately (models 3A–3D), the estimates of secondary human-to-human transmission are consistently much lower than the estimates of primary human-to-human transmission. These results suggest that humans infected by animals transmit the virus fairly efficiently to other humans, but that humans infected by other humans do not efficiently pass the virus on to other humans.

It is tempting to speculate that the observed difference between primary and secondary human-to-human transmission is due to a dose effect, i.e., that humans infected by humans had been infected with a lower number of virus particles than humans who were infected by poultry. Unfortunately, independent evidence of the “degree of infection” that could corroborate this suggestion is lacking. An alternative explanation that could conceivably explain the observed difference between primary and secondary human-to-human transmission would involve changes in behavior whereby people become more careful in preventing risky contacts after the index case shows signs of illness. Again, evidence supporting or against this hypothesis is lacking.

Often, it is assumed that the transmissibility of an emerging pathogen will increase as more individuals are infected, because it allows the pathogen to adapt to the new host species [20]. However, if anything, our analyses have shown that secondary human-to-human transmission is less efficient than primary human-to-human transmission, in contrast with conventional wisdom. A possibility that is invariably overlooked is that within-host selection of avian influenza viruses in human hosts does not select for higher but rather for lower transmissibility. Theoretical studies focusing on evolution of the dispersal rate in a metapopulation context indicate that this is a theoretical possibility [45]. Empirical studies with ferrets focusing on the transmissibility of H5N1 avian influenza viruses have begun to unravel the evolutionary pathways of these viruses [17–18], and indicate that evolutionary adaptation in mammals is a multifaceted process that is unlikely to lead to simple maximization of the transmission rate.

Our analyses indicate that the benefit of giving antiviral drugs to household members of an infected individual in terms of reducing the number of infections would have been modest. In fact, for the study population of Table 1, the results show that the number of household infections could have been decreased from more than 30 to 22–25 if antiviral drugs are moderately effective, and to 12–18 if antiviral drugs are more effective. This is due to the fact that antiviral drugs provide only partial protection against infection and shedding, while the estimate of the transmission rate of primary human-to-human transmission is relatively high. If the baseline transmission parameters are decreased, the potential effectiveness of antiviral treatment increases (unpublished data), in line with the observation that control measures usually are most effective whenever the basic reproduction number is close to 1 [9,38,43]. Alternatively, if the baseline transmission parameters are increased, the effectiveness of antiviral prophylaxis decreases.

Although our analyses suggest that antiviral drugs are only moderately effective in reducing the number of household infections, we do not intend to suggest that antiviral drug treatment should or should not be used as prophylaxis against avian influenza viruses. In fact, it may well be that even though prophylactic antiviral drug treatment is only moderately effective in preventing the number of human infections, it may still be quite effective in reducing the disease symptoms of individuals who are infected [40–41]. On the other hand, it is also possible that by reducing the disease symptoms, individuals who are infected will in effect be *more* infectious because they are less likely to remain bedridden. Studies quantifying the relation between disease and infectiousness (i.e., viral titers in the upper respiratory tract) are needed to answer this question.

Finally, with the number of H5N1 infections in humans accumulating at a steady pace, it is important to keep track of the ability of this virus to enhance its transmissibility in humans [7]. At present, however, there is still a conspicuous lack of data pertaining to the possibility of human-to-human spread of H5N1 viruses. In our opinion, detailed investigations of infected individuals, as well as tracing and investigation of the individuals in close contact with a confirmed case, should become an integral part of the handling of each human H5N1 infection.

## Supporting Information

Table S1 Overview of Parameter Estimates for the Models Described in Materials and Methods
(40 KB DOC)

Table S2 Overview of Parameter Estimates in Case of an Imperfect Serological Test (47 KB DOC)

Table S3 Overview of Parameter Estimates in the Case That Two Outlier Households Are Excluded from the Analyses (45 KB DOC)

Table S4 Overview of the Estimated Effectiveness of Antiviral Prophylaxis in Households of Size Four and Eight People (47 KB DOC)

Text S1 Supplementary Methods (66 KB DOC)
