# Supplementary material for: Detecting Emerging Transmissibility of Avian Influenza Virus in Human Households
Source: PLoS Comput Biol. 2007 Jul 27;3(7):e145. doi: 10.1371/journal.pcbi.0030145 (PMC1933478; doi:10.1371/journal.pcbi.0030145)
Supplement: Table S1 — (40 KB DOC) [file pcbi.0030145.st001.doc]

|  | model | | | parameter estimate  (95% CI) | Akaike information criterion (AIC_c_) | % support |
| --- | --- | --- | --- | --- | --- | --- |
| no secondary transmission | | 1A | β*_21_=0.890 (0.61-1.2) | | 57.15 | 8.5 |
|  |  | 1B | β*_21_=1.18 (0.64-2.3) | | 54.64 | 29.9 |
|  |  | 1C | β _21_=3.11 (2.1-4.3) | | 73.11 | <0.1 |
|  |  | 1D | β _21_=4.43 (2.4-8.5) | | 60.80 | 1.4 |
| equal primary and secondary transmission | | 2A | β*_22_=0.398 (0.27-0.56) | | 56.34 | 12.8 |
|  |  | 2B | β*_22_=0.506 (0.30-0.85) | | 59.21 | 3.1 |
|  |  | 2C | β _22_=1.73 (1.2-2.4) | | 57.23 | 8.2 |
|  |  | 2D | β _22_=2.12 (1.3-3.5) | | 59.27 | 2.9 |
| full model | | 3A | β*_21_=0.614 (0.34-1.0)  β*_22_=0.208 (0.0038-0.46) | | 55.54 | 19.1 |
|  |  | 3B | β*_21_=1.06 (0.46-2.2)  β*_22_=0.0714 (0-0.51) | | 56.81 | 10.2 |
|  |  | 3C | β_21_=1.70 (0.96-2.8)  β_22_=1.78 (0.78-3.1) | | 59.61 | 2.5 |
|  |  | 3D | β _21_=2.80 (1.3-6.7)  β_22_=1.33 (0-3.9) | | 60.92 | 1.3 |

Table S1. Maximum likelihood estimates of the transmission rate parameters for the models described in the Methods. Models ‘A’ and ‘B’ assume density-dependent transmission, and models ‘C’ and ‘D’ assume frequency-dependent transmission. Models ‘A’ and ‘C’ assume a fixed infectious period, and models ‘B’ and ‘D’ assume an exponentially distributed infectious period. Notice that the frequency-dependent models have low overall support.
