# Supplementary material for: Detecting Emerging Transmissibility of Avian Influenza Virus in Human Households
Source: PLoS Comput Biol. 2007 Jul 27;3(7):e145. doi: 10.1371/journal.pcbi.0030145 (PMC1933478; doi:10.1371/journal.pcbi.0030145)
Supplement: Table S2 — (47 KB DOC) [file pcbi.0030145.st002.doc]

|  | model | | | parameter estimate  (95% CI) | Akaike information criterion (AIC_c_) | AIC difference (Δ_i_) | % support |
| --- | --- | --- | --- | --- | --- | --- | --- |
| no secondary transmission | | 1A | β*_21_=0.727 (0.45-1.1) | | 57.15 | 2.780 | 5.3 |
|  |  | 1B | β*_21_=0.939 (0.47-1.9) | | 54.37 | 0 | 21.1 |
|  |  | 1C | β_21_=2.34 (1.4-3.6) | | 68.86 | 14.48 | <0.1 |
|  |  | 1D | β_21_=3.48 (0.47-1.9) | | 60.14 | 5.77 | 1.1 |
| equal primary and secondary transmission | | 2A | β*_22_=0.345 (0.22-0.51) | | 54.46 | 0.08 | 20.3 |
|  |  | 2B | β*_22_=0.445 (0.24-0.81) | | 55.31 | 0.93 | 13.3 |
|  |  | 2C | β_22_=1.53 (1.0-2.2) | | 56.50 | 2.12 | 7.3 |
|  |  | 2D | β_22_=1.90 (1.1-3.3) | | 56.52 | 2.15 | 7.2 |
| full model | | 3A | β*_21_=0.478 (0.22-0.87)  β*_22_=0.220 (0-0.52) | | 55.70 | 1.32 | 10.9 |
|  |  | 3B | β*_21_=0.734 (0.26-1.8)  β*_22_=0.140 (0-0.90) | | 56.28 | 1.91 | 8.2 |
|  |  | 3C | β_21_=1.28 (0.59-2.3)  β_22_=1.98 (0.76-4.1) | | 58.37 | 4.00 | 2.9 |
|  |  | 3D | β_21_=1.82 (0.68-4.6)  β_22_=2.03 (0-8.8) | | 58.90 | 4.53 | 2.2 |

Table S2. Maximum likelihood estimates of the transmission rate parameters for the models described in the Methods, assuming 85% specificity of the serological test that was used to confirm infection. Models ‘A’ and ‘B’ assume density-dependent transmission, and models ‘C’ and ‘D’ assume frequency-dependent transmission. Models ‘A’ and ‘C’ assume a fixed infectious period, and models ‘B’ and ‘D’ assume an exponentially distributed infectious period.
