# Supplementary material for: Detecting Emerging Transmissibility of Avian Influenza Virus in Human Households
Source: PLoS Comput Biol. 2007 Jul 27;3(7):e145. doi: 10.1371/journal.pcbi.0030145 (PMC1933478; doi:10.1371/journal.pcbi.0030145)
Supplement: Table S3 — (45 KB DOC) [file pcbi.0030145.st003.doc]

|  | model | | | parameter estimate  (95% CI) | Akaike information criterion (AIC_c_) | AIC difference (Δ_i_) | % support |
| --- | --- | --- | --- | --- | --- | --- | --- |
| no secondary transmission | | 1A | β*_21_=0.765 (0.49-1.1) | | 47.84 | 0.97 | 14.3 |
|  |  | 1B | β*_21_=1.02 (0.52-2.1) | | 46.88 | 0 | 23.3 |
|  |  | 1C | β_21_=2.36 (1.5-3.5) | | 54.74 | 7.86 | 0.5 |
|  |  | 1D | β_21_=3.25 (1.7-6.5) | | 50.13 | 3.25 | 4.6 |
| equal primary and secondary transmission | | 2A | β*_22_=0.443 (0.28-0.66) | | 47.56 | 0.68 | 16.6 |
|  |  | 2B | β*_22_=0.551 (0.30-1.0) | | 50.04 | 3.17 | 4.8 |
|  |  | 2C | β_22_=1.55 (1.0-2.3) | | 48.68 | 1.80 | 9.5 |
|  |  | 2D | β_22_=1.91 (1.1-3.4) | | 50.33 | 3.45 | 4.1 |
| full model | | 3A | β*_21_=0.591 (0.31-1.0)  β*_22_=0.227 (0-0.64) | | 48.43 | 1.56 | 10.7 |
|  |  | 3B | β*_21_=0.988 (0.41-2.1)  β*_22_=0.0265 (0-0.70) | | 49.29 | 2.41 | 7.0 |
|  |  | 3C | β_21_=1.60 (0.86-2.7)  β_22_=1.46 (0.26-3.2) | | 51.09 | 4.21 | 2.8 |
|  |  | 3D | β_21_=2.58 (1.1-6.1)  β_22_=0.793 (0-4.1) | | 51.85 | 4.97 | 1.9 |

Table S3. Maximum likelihood estimates of the transmission rate parameters for the models described in the Methods if the two large households in Table 1 are excluded from the analyses. Models ‘A’ and ‘B’ assume density-dependent transmission, and models ‘C’ and ‘D’ assume frequency-dependent transmission. Models ‘A’ and ‘C’ assume a fixed infectious period, and models ‘B’ and ‘D’ assume an exponentially distributed infectious period.
