# Supplementary material for: Detecting Emerging Transmissibility of Avian Influenza Virus in Human Households
Source: PLoS Comput Biol. 2007 Jul 27;3(7):e145. doi: 10.1371/journal.pcbi.0030145 (PMC1933478; doi:10.1371/journal.pcbi.0030145)
Supplement: Table S4 — (47 KB DOC) [file pcbi.0030145.st004.doc]

|  | model | | no treatment | | antiviral treatment 1 | | antiviral treatment 2 | |
| --- | --- | --- | --- | --- | --- | --- | --- | --- |
|  |  |  | N=4 | N=8 | N=4 | N=8 | N=4 | N=8 |
| no secondary transmission | | 1A | 1.77 (0.07) | 4.13 (0.00) | 1.39 (0.15) | 3.25 (0.13) | 0.90 (0.34) | 2.10 (0.08) |
|  |  | **1B** | **1.62 (0.22)** | **3.79 (0.11)** | **1.36 (0.29)** | **3.17 (0.15)** | **0.96 (0.41)** | **2.24 (0.23)** |
|  |  | 1C | 1.62 (0.10) | 2.26 (0.07) | 1.26 (0.20) | 1.67 (0.15) | 0.80 (0.39) | 1.01 (0.34) |
|  |  | 1D | 1.58 (0.23) | 2.49 (0.21) | 1.31 (0.30) | 1.96 (0.27) | 0.92 (0.43) | 1.27 (0.39) |
| equal primary and secondary transmission | | **2A** | **1.46 (0.30)** | **5.88 (0.06)** | **1.03 (0.43)** | **4.47 (0.14)** | **0.56 (0.62)** | **2.26 (0.33)** |
|  |  | 2B | 1.36 (0.40) | 4.72 (0.22) | 1.04 (0.48) | 3.80 (0.29) | 0.63 (0.62) | 2.31 (0.41) |
|  |  | 2C | 1.58 (0.27) | 3.39 (0.22) | 1.12 (0.40) | 2.11 (0.35) | 0.61 (0.59) | 0.95 (0.54) |
|  |  | 2D | 1.40 (0.39) | 3.01 (0.35) | 1.08 (0.47) | 2.11 (0.43) | 0.66 (0.61) | 1.10 (0.57) |
| full model | | **3A** | **1.67 (0.16)** | **5.36 (0.01)** | **1.13 (0.28)** | **3.05 (0.05)** | **0.68 (0.48)** | **1.74 (0.18)** |
|  |  | **3B** | **1.61 (0.24)** | **4.08 (0.12)** | **1.30 (0.31)** | **3.12 (0.16)** | **0.90 (0.44)** | **2.15 (0.25)** |
|  |  | 3C | 1.57 (0.28) | 3.40 (0.23) | 0.91 (0.41) | 1.35 (0.35) | 0.52 (0.60) | 0.68 (0.55) |
|  |  | 3D | 1.50 (0.32) | 2.93 (0.29) | 1.07 (0.41) | 1.65 (0.37) | 0.69 (0.54) | 0.95 (0.50) |

Table S4. The expected outbreak size and probability of no household transmission (number between brackets) in households of sizes four (N=4) and eight (N=8), assuming variable efficacy of antiviral treatment. Parameter values of the transmission parameters are as in Table S1. Models with at least 10% support are in boldface. The antiviral efficacies for susceptibility (*AVE_S_*) and infectiousness (*AVE_I_*) are set at *AVE_S_=0.3* and *AVE_I_=0.6* in the columns under the heading ‘antiviral treatment 1’ (see Table 3), and *AVE_S_=0.6* and *AVE_I_=0.66* in the columns under the heading ‘antiviral treatment 2’.
