# Supplementary material for: Detecting Emerging Transmissibility of Avian Influenza Virus in Human Households
Source: PLoS Comput Biol. 2007 Jul 27;3(7):e145. doi: 10.1371/journal.pcbi.0030145 (PMC1933478; doi:10.1371/journal.pcbi.0030145)
Supplement: Text S1 — (66 KB DOC) [file pcbi.0030145.sd001.doc]

Detecting emerging transmissibility of avian influenza virus

in human households

Text S1

**Model Structure**

The Methods section of the main text gives an overview of our methodology. Here we give a number of additional technical details. Reference numbers refer to the references in the main text.

The analyses are based on a stochastic SEIR epidemiological model in which individuals are classified as susceptible (S), latently infected (infected but not yet infectious)(E), infectious (I), and recovered and immune (R). No a priori assumptions are made regarding the duration or distribution of the latent and infectious periods. We consider a model with two types of individuals, where *n_i_* and *a_i_* represent the initial number of susceptible and infected type *i* individuals in a household, respectively (*i=1,2*). In the following, type 1 individuals are assumed to have been infected by animals, while type 2 individuals have been infected by humans. There are no individuals that have prior immunity, so that total household size is given by *N=n_1_+n_2_+a_1_+a_2_*. The parameter *B_i_* denotes the probability that a type *i* (*i=1,2*) individual escapes infection from outside the household (i.e. from the animal reservoir). The final size distribution is determined by triangular equations that can be solved recursively [34-35]:

(A1)

(*j_1_=0,1,…, n_1_, j_2_=0,1,…, n_2_*). The factor in the numerator of equation (A1) represents the probability that$\omega_{1}$type 1 and$\omega_{2}$type 2 individuals are infected in a population with *j_1_* and *j_2_* susceptibles, while $\prod_{k=1}^{2} B_{k}^{n_{k}-j_{k}}\phi_{k}\left[\sum_{l=1}^{2} \beta^{*}_{lk}\left(n_{l}-j_{l}\right)\right]^{ \omega_{k}+a_{k}}$ in the denominator is the probability that all initially present susceptible individuals avoid infection. For given *n_i_* and *a_i_* the final size distribution is fully specified by the escape probabilities$B_{i}$, the Laplace transforms $\phi_{i}\left[s\right]$ of the infectious period probability distributions, and the transmission parameters $\beta_{i j}^{*}$ ($i,j=1, 2$).

**Scenarios**

Equation (A1) is quite general and can be used when only limited information on the nature of the transmission chain in the households is available. The data of the Dutch epidemiological study are more specific and allow a number of simplifications (see Methods for details). In particular, in our analyses we have throughout taken *B_2_=1*, *a_1_=1*, *a_2_=0*, and *n_1_=0*.

Four scenarios are considered that are defined by the assumptions regarding the distribution of the infectious period and the mechanism of pathogen transmission. With respect to the infectious period we assume that the infectious period is exponentially distributed (the ‘general stochastic epidemic’) or of fixed duration (the ‘Reed-Frost’ model). If the infectious period is exponentially distributed, the Laplace transform of the infectious period probability distribution is given by $\phi\left[s\right]=1/\left(1+Ts\right)$. If, on the other hand, the infectious period is of fixed duration it is given by $\phi\left[s\right]=\exp\left[-Ts\right]$. For simplicity we assume that the infectious period is independent of the source of infection, i.e. $\phi_{1}\left[s\right]=\phi_{2}\left[s\right]=\phi\left[s\right]$. Further, by rescaling the time axis we may measure time in units of the infectious period [31-32]. Without loss of generality we may therefore take $T=1$.

Furthermore, we assume that transmission is frequency-dependent or density-dependent [37]. In a frequency-dependent model the number of contacts per unit of time is fixed, and the transmission rate is proportional to the relative frequency (prevalence) of infectious individuals. In a density-dependent model the number of contacts per unit of time is proportional to the number of individuals. Equation (A1) describes a density-dependent transmission process. In a frequency-dependent transmission model the argument of $\phi_{k}$ in equation (A1) is divided by household size (*N*), i.e. $\beta_{l k}^{*}\left(n_{l}-j_{l}\right)$ has to be replaced by $\frac{\beta_{l k}\left(n_{l}-j_{l}\right)}{N}$. Hence, the transmission parameters of the density-dependent model are denoted by $\beta_{i j}^{*}$ and those of the frequency-dependent model are given by $\beta_{i j}$($i,j=1, 2$).

Notice that if the infectious period is fixed the distribution of the number of type *i* infections caused by a type *j* infected individual in a large susceptible population (the ‘offspring distribution’) would be Poisson with mean $\beta_{ij}$ or $\beta_{ij}^{*}N$(depending on whether transmission is frequency- or density-dependent). If, on the other hand, the infectious period is exponentially distributed, there is more variation in the number of infections caused by an infected individual. In this case, the number of infections caused by an infected individual in a large susceptible population has a geometric distribution with parameter $\frac{1}{1+\beta_{ij}}$ or $\frac{1}{1+\beta_{ij}^{*}N}$ [36].

Notice furthermore that if secondary human-to-human transmission is absent (model 1 in Tables 2-3 and Tables S1-S4), the household final size is binomially distributed if the infectious period is of fixed duration (with parameter $\frac{\beta}{1+\beta}$ and binomial totals given by the number of susceptibles, $n_{2}$). If, on the other hand, the infectious period is exponentially distributed, then the final size is given by a compound exponential-binomial distribution. In case of the former model assumption the final size distribution is clearly unimodal, while for the latter simulations also indicate that the final size distribution remains unimodal.

In Tables S1-S4 we focus on scenarios that are defined by the mechanism of pathogen transmission (density-dependent versus frequency-dependent), and by the distribution of the infectious period (fixed versus exponentially distributed). Throughout, models denoted by ‘A’ and ‘B’ assume density-dependent transmission, while models ‘C’ and ‘D’ assume frequency-dependent transmission. Furthermore, models denoted by ‘A’ and ‘C’ assume a fixed infectious period, while models ‘B’ and ‘D’ assume an exponentially distributed infectious period. Hence, model ‘A’ assumes density-dependent transmission and a fixed infectious period, model ‘B’ assumes density-dependent transmission and an exponentially distributed infectious period, and so forth.
